# Whole Body Vibration Training Improves Maximal Strength of the Knee Extensors, Time-to-Exhaustion and Attenuates Neuromuscular Fatigue

**DOI:** 10.3390/sports11050094

**Published:** 2023-04-25

**Authors:** Serge S. Colson, Jennifer Gioda, Flavio Da Silva

**Affiliations:** Université Côte d’Azur, LAMHESS, France; jennifer.gioda@univ-cotedazur.fr (J.G.); flavio.da-silva@univ-cotedazur.fr (F.D.S.)

**Keywords:** voluntary activation, evoked contractions, knee flexors, central factors, peripheral factors

## Abstract

Whole-body vibration (WBV) training programs were reported to improve knee extensor muscle (KE) strength in healthy participants. Unfortunately, the underlying mechanisms of these strength gains remain unresolved. In addition, WBV training was shown to increase the time-to-exhaustion of a static submaximal endurance task. However, the effects of WBV training on neuromuscular fatigue (i.e., a decrease of the maximal voluntary isometric contraction; MVIC) induced by an endurance task is unknown. We, therefore, investigated the influence of WBV training on (i) KE MVIC and neuromuscular function, (ii) the time-to-exhaustion of the KE associated with a submaximal isometric fatiguing exercise, and (iii) KE neuromuscular fatigue and its etiology. Eighteen physically active males were assigned to a WBV group (*n* = 10) or a sham training group (SHAM; *n* = 8). The MVIC of the KE, voluntary activation, and electrically evoked responses of the KE were assessed (i) before and after a fatiguing exercise (i.e., submaximal isometric contraction) performed until failure, and (ii) before (PRE) and after a 6-week training (POST) period. At POST, the WBV training increased the KE MVIC (+12%, *p* = 0.001) and voluntary activation (+6%, *p* < 0.05) regardless of the fatiguing exercise. The time-to-exhaustion was also lengthened at POST in the WBV group (+34%, *p* < 0.001). Finally, the relative percentage of MVIC decrease after fatiguing exercises diminished in the WBV group between PRE and POST (−14% vs. −6%, respectively, *p* < 0.001). Significant neural adaptation enhancements account for the trend in KE strength improvements observed after the WBV training program. In addition, the WBV training was effective at increasing the time-to-exhaustion and attenuating neuromuscular fatigue.

## 1. Introduction

Over the past two decades, whole body vibration (WBV) training has been widely used to enhance maximal muscle strength [1,2,3,4,5,6]. Recent meta-analyses have shown that WBV training was more effective than the same training program without WBV to improve maximal strength of the knee extensor muscles in healthy participants [7] and in competitive/elite athletes [8]. Notwithstanding the knee extensors’ strength gains observed after WBV training, the underlying mechanisms behind these gains are yet to be identified. To date, although enhanced electrically evoked muscle responses [4] and increased muscle thickness [9] were reported after the WBV training programs, no difference was observed with the same training programs realized without WBV. Neural adaptations, which are often invoked as candidate mechanisms [10], are still a matter of debate due to the inconsistent observations reported. Most studies failed to observe greater improvements in voluntary activation [3,4,11], corticospinal excitability [9,12], or spinal reflexes [13] after WBV training in comparison to an identical training program without WBV. Consequently, the underlying mechanisms explaining maximal strength increases of the knee extensor muscles after WBV training in healthy participants still need to be elucidated.

While most studies have examined the effects of WBV training on strength production [1,2,3,4,5,6,9,11,12], no study has yet investigated the WBV training effects on neuromuscular fatigue in healthy participants. During physical exercise, neuromuscular fatigue gradually develops because of the repetition and/or maintenance of muscle contractions [14]. Neuromuscular fatigue is commonly defined as a decline in performance (e.g., the decreased maximal muscle force) of the exercised muscle, which involves complex interactions between the peripheral and central factors [14]. Peripheral factors refer to muscle contractile function perturbations evidenced by impaired electrically-evoked muscle responses [15], whereas central factors are attributed to adjustments within the central nervous system at the spinal and/or supraspinal levels [14].

After a single session of WBV training, even though some studies have shown that neuromuscular fatigue occurred in the knee extensor muscles, no difference was observed with the same session performed without WBV [16,17,18,19]. In these studies, the central factors, assessed by the measure of voluntary activation, did not change [16,17,18,19]. In contrast, the peripheral factors, assessed via electrically evoked muscle responses, were impaired [17,18,19], but no difference was demonstrated between the WBV and non-WBV sessions. In healthy participants, the influence of a single WBV session in comparison to a non-WBV session on neuromuscular fatigue is unclear and the effects of WBV training on neuromuscular fatigue are unknown.

To the best of our knowledge, only one study has demonstrated that a 4-week WBV training program improved muscle endurance, assessed by a static endurance task of the lower limbs performed until failure [20]. However, using such submaximal sustained isometric contractions only provides an index of fatigue performance (i.e., endurance time or time-to-exhaustion), and no information regarding the decline in maximal voluntary isometric contraction induced by the task (i.e., neuromuscular fatigue), nor were the peripheral and central contributions obtained. To date, one animal study showed evidence that the force generated by the fast, fatigue-resistant motor units of a rat’s medial gastrocnemius muscles declined less and was better maintained during a fatigue test after three months of WBV training [21]. Although this interesting observation could suggest that neuromuscular fatigue would be reduced after a WBV training program, it has never been investigated in healthy human participants.

The purpose of the present study was to investigate the effects of WBV training of the knee extensor muscles on (i) the maximal voluntary isometric force production, (ii) the time-to-exhaustion of a submaximal isometric fatiguing exercise, and (iii) that neuromuscular fatigue and its etiology. Although no hypothesis could be formulated regarding the peripheral and central factors involved in the maximal voluntary isometric strength production adaptations after WBV training, due to inconsistent observations, knee extensor strength gains were expected [1,3,5,6]. In addition, based on human [20] and animal [21] observations, it was hypothesized that the time-to-exhaustion would increase along with less maximal voluntary isometric contraction reduction induced by the fatiguing exercise after the WBV training.

## 2. Materials and Methods

### 2.1. Participants

Based on a previous study evidencing the improvements of the maximal voluntary isometric contraction in the knee extensor muscles, after an 8-week WBV training period performed at a vibration frequency of 30 Hz [6], the minimum sample size was estimated a priori with G*Power (v.3.1.9.6). For the repeated measures within-between the participant analyses with a power set at 0.95, fourteen participants had to be included. For the sample size of the participants included in the present study, a sensitivity power analysis led to a large effect of *f* = 0.45 (i.e., α = 0.05 and power = 0.95). Eighteen healthy active male participants volunteered to participate in this study. They were randomly assigned to a WBV training group (WBV; *n* = 10; age: 21.7 ± 3.2 years, height: 180 ± 5.4 cm, body mass: 75.3 ± 7.4 kg; mean ± SD) or a sham training group (SHAM; *n* = 8; age: 21.5 ± 3.5, height: 179.1 ± 7.3, body mass: 70.7 ± 8.5 kg; mean ± SD). All participants were physically active and were regularly trained in various physical activities. Based on self-reports, the average weekly exercising physical activity time was 10.8 ± 5.7 and 9.8 ± 3.2 h for WBV and SHAM, respectively. None of them had engaged in WBV training programs in the 3 months preceding the beginning of the experiments, nor had they previously experienced WBV exercise as a training modality. Participants were asked to refrain from strenuous physical activity for 24 h prior to each testing session and to maintain their normal physical activity levels during the experimental period. The local research ethics committee of the University of Nice Sophia Antipolis approved the study (2008-003) and all procedures were in conformity with the latest version of the Declaration of Helsinki (2013). All participants were informed of the purpose, procedures, and potential risks of the study and gave informed written consent prior to enrollment in the investigation.

### 2.2. Experimental Procedures

**Figure 1:**
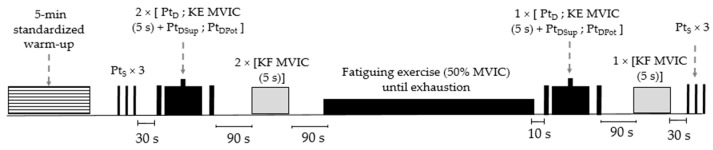
Overview of the experimental procedure performed before and after the training period. Pt_S_ Maximal peak twitch at rest (1 Hz), KE Knee extensor muscles, MVIC Maximal voluntary isometric contraction, Pt_D_ Maximal peak twitch at rest (100 Hz), Pt_DSup_ Superimposed peak twitch (100 Hz), Pt_DPot_ Potentiated peak twitch (100 Hz), KF Knee flexor muscles.

The experimental procedure consisted of two testing sessions realized at a baseline (PRE) and after (POST) a 6-week training period. A week before the baseline, an initial session familiarized the participants with the equipment and testing procedures. At each testing session, surface electromyography (sEMG) electrodes were placed on the participants (see sEMG recordings for details) upon arrival, followed by a 5 min standardized warm-up on a cycling ergometer (Monark, 818E, Varberg, Sweden) at a pedaling frequency of 70 revs.min^−1^, with an intensity set at 2 watts.kg^−1^. Then, they sat on an isokinetic dynamometer, after having determined the stimulation site and the intensity of the percutaneous electrical stimulation, the neuromuscular function of the right knee extensor muscles (KE) was assessed (for details see Neuromuscular function evaluation and Percutaneous nerve stimulation). Three single stimulations (rest interval: 5 s) were delivered at rest, followed by two 5 s maximal voluntary isometric contractions (MVIC) of the KE (rest interval: 90 s) with doublet stimulations delivered 2 s before, during, and 4 s after the contractions, respectively. After 90 s of rest, two maximal voluntary isometric knee flexions were performed (rest interval: 90 s). The procedure was repeated immediately after a submaximal fatiguing exercise (see Fatiguing exercise for details); participants carried out one MVIC with electrical stimulation followed by one maximal voluntary isometric knee flexion and then three single stimulations (Figure 1).

### 2.3. Fatiguing Exercise

At each testing session (PRE and POST), the fatiguing exercise consisted of a submaximal isometric knee extension of the right lower limb maintained until exhaustion at a 50% target value of the MVIC of the day [22]. Automatic visual feedback of the isometric torque exerted and the 50% MVIC target to reach was displayed on the dynamometer screen. The fatiguing exercise ended when the torque declined under the 50% MVIC target for more than three seconds (despite strong verbal encouragement). The time elapsed from the beginning of the exercise was recorded as time-to-exhaustion. Participants were not informed of their time-to-exhaustion until the completion of the POST testing session.

### 2.4. Neuromuscular Function Evaluation

Voluntary and electrically induced contractions were measured using an isokinetic dynamometer (Biodex^TM^, System 3, BIODEX Corporation, Shirley, NY, USA). Participants were placed in a seated position with a trunk-thigh angle of 110° and the knee flexed at 60° (0° = full knee extension). The axis of the dynamometer was aligned with the anatomical knee flexion-extension axis, and the dynamometer lever arm was strapped to the lower leg, 1 cm above the external malleolus. Each participant was securely strapped to the dynamometer chair with belts over the hips and chest. Participants kept their arms crossed on their chest during the testing procedure. The torque signal from the dynamometer was collected with software (Acqknowledge 3.8.2, Biopac^®^ Systems Inc., Holliston, MA, USA). The maximal voluntary isometric torques were computed as the mean value of the torque signal over a 500 ms window over the highest torque value. The best MVIC and maximal voluntary isometric knee flexion were analyzed before the fatiguing exercise.

### 2.5. Percutaneous Nerve Stimulation

Electrically evoked contractions of the KE were induced by femoral nerve stimulation. Rectangular impulses (maximum voltage: 400 V; duration: 2 ms) were delivered using a constant-current stimulator (model DS7, Digitimer^®^, Hertfordshire, UK). Evoked contractions were elicited by the same experimenter using a monopolar ball electrode (10 mm diameter) pressed on the femoral triangle, 3–5 cm below the inguinal ligament. The anode was a large electrode (50 × 90 mm; Stimex^®^, Wetzlar, Germany) located at the level of the great trochanter. The individual’s stimulation intensity, determined by relaxed muscles, was increased progressively until no further concomitant increase in the mechanical peak twitch torque (Pt_S_) and peak-to-peak electrophysiological response (M_max_) were observed. To ensure the adequate assessment of the KE neuromuscular function, stimulation intensity was increased further by 20%. Individual supramaximal intensities ranged between 60 and 160 mA. Doublet stimuli (100 Hz) were delivered before (Pt_D_), during (Pt_DSup_), and after (Pt_DPot_) the MVIC. The maximal peak twitch amplitudes of Pt_S_ (average of the three), Pt_D_, and Pt_DPot_ were retained for the analyses. Post-contraction potentiation (PCP) was computed from the Pt_DPot_-Pt_D_ ratio. The voluntary activation level (VA) of the KE was calculated using the following formula [23]:

$$VA\left(\%\right)=\left(1-\left(\frac{Pt_{DSup}}{Pt_{DPot}}\right)\right)\times 100$$

### 2.6. sEMG Recordings

The sEMG signals from the rectus femoris (RF), vastus medialis (VM), and vastus lateralis (VL) muscles of the right limb were recorded with pairs of Ag/AgCl electrodes (1 cm diameter; Contrôle Graphique Medical^®^, Brie-Comte-Robert, France). The electrodes were fixed lengthwise over the muscles (inter-electrode distance = 2 cm) according to the SENIAM recommendations [24]. A reference electrode was positioned on the external condyle of the left knee. The low impedance between the pairs of electrodes (<5 kΩ) was obtained by shaving, abrading, and cleaning the skin. Each electrode placement was marked on the skin to reproduce identical repositioning between the PRE and POST testing sessions. Electrodes were secured to the skin with medical tape (Tegaderm™ Film, 3M™, Cergy-Pontoise, France). The sEMG signals were amplified (MP100 Biopac^®^ Systems Inc., Holliston, MA, USA; CMRR = 110 db, Z input = 1000 MΩ, gain = 1000), filtered (bandwidth frequency = 10 Hz to 500 Hz), and simultaneously recorded (sampling frequency, 2 kHz) using the Acknowledge software previously mentioned. The M_max_ peak-to-peak amplitudes (average of the three responses) were analyzed for the RF, VM, and VL muscles. The root mean square (RMS) values of KE sEMG were computed over the 500 ms period of the corresponding torque analysis during the MVIC and maximal voluntary isometric knee flexion measures. The RMS values of RF, VM, and VL were normalized to the respective M_max_ (i.e., RMS/M_max_) measured before and after the fatiguing exercise, and for each testing session (PRE and POST), the sum of the KE RMS/M_max_ values was computed.

### 2.7. WBV and SHAM Training

Over a 6-week period, participants performed three sessions of 20 min per week of unloaded static exercises on a synchronous vertical WBV platform (Silverplate, 100 × 65 cm, Silver^®^ Développement, Valbonne, France). Prior to each training session, a 3 min standardized warm-up consisting of 60 complete knee flexion extensions was performed. Participants had to maintain isometric squatting positions lasting 30 s, immediately followed by resting periods of 30 s (duty cycle: 50%; 10 repetitions; total duration of vibration exposure: 10 min). Participants positioned their hands on their waist so no external postural support was provided and they tilted their trunks slightly forward. A relaxed standing position was adopted during the 30 s rest periods. The participants stood on a 20-mm closed-cell expanded rubber mat (compression deflection 1.2 kg cm^−2^, compression set ≤ 80%, tearing resistance ≥ 0.5 kN m^−1^; Silver^®^ Développement, Valbonne, France) and wore socks during training sessions. During the WBV sessions, the vibration frequency and peak-to-peak displacement were set at 30 Hz (i.e., constant frequency) and 4 mm, respectively (i.e., manufacturer specifications; theoretical peak acceleration = 7.25 g). The effective acceleration delivered by the platform was quantified with a tri-axial accelerometer (50G, TSD109F, Biopac^®^ Systems, Holliston, MA, USA) placed on the center of the platform without participants. The effective acceleration measured was 3.58 g and 3.33 g with and without the use of the rubber mat, respectively. The rubber mat did not influence the effective frequency delivery of 33.07 Hz. Peak-to-peak displacements were computed by double integration of the platform acceleration signal, which resulted in 1.63 mm and 1.51 mm with and without the use of the rubber mat, respectively. Similar theoretical WBV settings (i.e., acceleration, frequency, and peak-to-peak displacement) have been successfully used to increase isometric KE strength [6]. Participants included in the SHAM group stood on the platform without vibration and followed the exact same training as the WBV group.

### 2.8. Statistical Analysis

Statistical analyses were performed using IBM SPSS Statistics software (IBM Corp., version 25 for Windows, Armonk, NY, USA). The influence of the WBV and SHAM training programs and muscle fatigue on the different variables measured was studied using generalized linear mixed-effect models (GLMMs) with a linear distribution. GLMMs were tested with and without the inclusion of a random effect (i.e., participant) using the Akaike information criterion (AIC). The model with the lowest AIC score was kept for analysis [25]. The GLMMs included the group (WBV vs. SHAM), training (PRE vs. POST), and fatigue (before vs. after) fixed effects for the MVIC, VA, the sum of the KE RMS/M_max_ values, M_max_ values, Pt_S_ Pt_D_, Pt_Dpot_, and maximal voluntary isometric knee flexion. Time-to-exhaustion and 50% of MVIC targets were examined based on the group (WBV vs. SHAM) and training (PRE vs. POST) effects. When a significant interaction or main effect was noted, Bonferroni-adjusted pairwise comparisons were used and the significance was set to *p* < 0.05. The meaningfulness of the significant results was estimated by Cohen’s d-effect size (ES). Unless specified, the data is expressed as mean ± SD (standard deviation) in the manuscript, table, and figures.

## 3. Results

### 3.1. Effects of the Training Period on Neuromuscular Function

A significant main effect of training (F_(1,64)_ = 7.28; *p* = 0.009) was observed for the MVIC of the KE as well as a tendency for a significant group × training interaction (F_(1,64)_ = 3.38; *p* = 0.071). Regardless of the group and fatigue measures, the MVIC of the KE increased from PRE to POST training (+7.83 ± 16.07%; *p* = 0.009; Table 1). Considering the tendency revealed by the GLMM analysis, pairwise comparisons were analyzed and indicated that the KE MVIC was significantly improved at POST (pooled MVIC values measured before and after the fatiguing exercise) in the WBV group (+11.95 ± 18.62%; *p* = 0.001; ES = 0.55; Table 1), but not in the SHAM group (+2.67 ± 10.60%; *p* = 0.567; Table 1). A significant group × training interaction was also highlighted for the maximal voluntary isometric knee flexion (F_(1,64)_ = 5.72; *p* = 0.020). Regardless of fatigue measures, in the WBV group, maximal voluntary isometric knee flexion measured at POST increased (+22.69 ± 23.50%; *p* < 0.001; ES = 0.56; Table 1; pooled values measured before and after the fatiguing exercise). Regardless of fatigue measures, voluntary activation showed a significant group × training interaction (F_(1,64)_ = 6.85; *p* = 0.011), with enhancements at POST in the WBV group (+6.37 ± 11.60%; *p* = 0.023; ES = 0.80; Table 1; pooled VA values measured before and after the fatiguing exercise) but not in the SHAM group (−3.28 ± 10.30%; *p* = 0.158). No significant training changes were evidenced for the KE RMS/M_max_ values (Table 1), M_max_, Pt_S_, Pt_D_, Pt_DPot_, and PCP values (Table 2).

### 3.2. Effects of the Fatiguing Exercise on Neuromuscular Function

A significant main effect of the fatiguing exercise (F_(1,64)_ = 14.78; *p* < 0.001) was observed for the KE MVIC and for PCP (F_(1,64)_ = 16.97; *p* < 0.001). Regardless of the group and training, the KE MVIC and PCP were impaired after the fatiguing exercise (−10.32 ± 13.21%; and −8.54 ± 12.01%, respectively). The sum of the KE RMS/M_max_ displayed a significant group × fatigue interaction (F_(1,64)_ = 8.78; *p* = 0.004), with decreased values after the fatiguing exercise in the WBV group (−17.03 ± 17.94%; *p* = 0.005; ES = 0.48), regardless of training measurements. No significant fatiguing exercise effects were reported for the VA (Table 1), M_max_, Pt_S_, Pt_D_, Pt_DPot_ (Table 2), and maximal voluntary isometric knee flexion.

### 3.3. Effects of the Training Period on the Fatiguing Exercise

**Figure 2:**
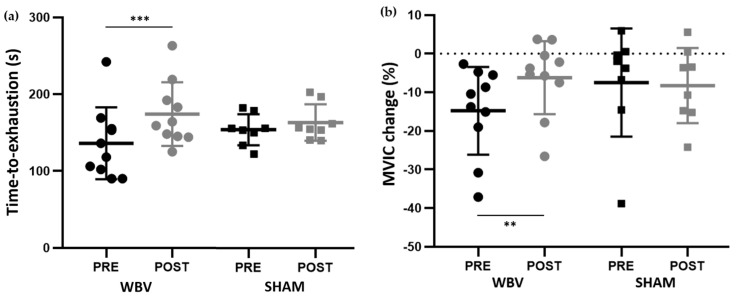
Data of the submaximal fatiguing exercise performed at 50% of MVIC measured before (PRE, black) and after (POST, gray) the 6-week training period for WBV (circles) and SHAM (squares) groups. Panel (**a**): Time-to-exhaustion. Panel (**b**): Relative percentage of MVIC change after the fatiguing exercise. ** and *** significant difference between PRE and POST at *p* < 0.01 and *p* < 0.001, respectively for the WBV group. MVIC Maximal voluntary isometric contraction.

A significant group × training interaction (F_(1,32)_ = 7.01; *p* = 0.012) was noted for time-to-exhaustion duration whereas the target value of 50% MVIC did not change (F_(1,32)_ = 0.28; *p* = 0.598). The Time-to-exhaustion of the WBV group was longer (+38.1 ± 29.8 s) after the training period (+34.04 ± 30.40%; *p* < 0.001; ES = 0.81; Figure 2a).

The analysis of the relative percentage of the MVIC decrease also revealed a significant group × training interaction (F_(1,32)_ = 4.73; *p* = 0.037). Pairwise comparisons also revealed that the percentage of the MVIC decrease after the fatiguing exercise was smaller at POST compared to PRE (i.e., −6.21 ± 9.45% vs. −14.78 ± 11.38%, *p* = 0.005; ES = 0.77; Figure 2b) in the WBV group only.

## 4. Discussion

Although WBV has been extensively used for strength training purposes, the effects of WBV training on the underlying mechanisms of strength adaptations is still a matter of debate in the literature, and the influence of WBV training on neuromuscular fatigue is unknown. In the present study, we observed, for the first time, that central factors (i.e., enhanced voluntary activation) contributed to the trend for the KE MVIC to increase after the 6-week WBV training period. Second, the time-to-exhaustion of the KE measured during a submaximal isometric exercise was longer after the 6-week WBV training program, whereas the performance of the SHAM group did not change. Third, this is the first study to report that KE neuromuscular fatigue (i.e., MVIC decrease after the fatiguing exercise) was reduced after the WBV training only. Fourth, WBV training also enhanced maximal voluntary isometric knee flexion strength.

Regarding the influence of the 6-week training period, the MVIC of the KE tended to improve significantly after WBV training in comparison to the SHAM training. Previous studies using synchronous vertical WBV platforms have evidenced significant improvements in KE isometric strength ranging from 16.6% to 36.8% after WBV training programs lasting between 7 to 12 weeks [1,5,6]. Regardless of the WBV settings (i.e., frequency, amplitude, and acceleration of the platform) or the WBV exercise performed (e.g., sets, repetitions, positions, or movements performed, exercise duration, and rest periods), it is worth mentioning that mainly untrained, healthy females were enrolled in these previous studies. In contrast, in another 6-week WBV study performed on a synchronous vertical platform, no significant KE isometric strength improvements of the physically active male participants were reported between WBV and SHAM training groups [4]. Other WBV studies of similar duration performed on side-alternating platforms, including mainly male participants, did not evidence isometric strength improvements of the KE [2,3]. Taken together, it seems that the participant characteristics (e.g., sex, fitness level, or training history) may account for the ~12% MVIC increase tendency observed here. Indeed, in WBV studies including active healthy males [2,3,4], similar to the present investigation, KE isometric strength of the participants was not increased significantly, whereas such improvements were evidenced in untrained healthy females studies [1,5,6]. We assume that the ~3.4 g stimulus of our WBV training sessions may have not been sufficient to significantly improve the KE MVIC of our physically active male participants. Consequently, the effects of the WBV resistance training on the KE isometric strength enhancements in male participants still needs extensive investigation.

To delineate the peripheral and central factors that contribute to the KE strength enhancements after WBV training, electrically evoked muscle responses, sEMG, and voluntary activation of the KE were assessed. In our study, no modification of the electrically evoked muscle responses (Pt_S_, Pt_D_, and Pt_DPot_), M_max_ values, nor the sEMG values of the KE were noted. Although the Pt_S_ and Pt_D_ were previously reported to increase, these improvements were no different between the WBV and SHAM training groups [4]. The absence of a modification of the other variables (M_max_ and sEMG values of the KE) is in line with the previous literature [4]. In contrast, we observed that voluntary activation was significantly increased (+6.7%; pooled values before and after the fatiguing exercise) after the 6-week WBV training. This result contradicts the findings from the previous studies where voluntary activation was unchanged following the WBV training over periods ranging from 2 to 11 weeks [2,4,11]. Although a side-alternating platform was used in two of these studies [2,11], male participants from the third study were trained on a synchronous vertical platform [4]. In this latter study, also lasting 6 weeks, the male participants had similar characteristics (e.g., sex, age, training background, etc.) and similar neuromuscular function attributes (i.e., KE MVIC, voluntary activation) as those in the present study. Only the frequency and peak-to-peak displacement combinations of the WBV training programs were different from our study, but it is extremely challenging to speculate on these parameters considering the absence of an effective measurement of the platform acceleration in Petit et al.’s study [4]. For these reasons, recommendations have been formulated when implementing and reporting the WBV studies [26,27,28,29], although many studies still fail to report the appropriate information. Therefore, we suggest that the ~3.4 g stimulus of our WBV training sessions may have “maximized” the individual muscle activation response [30] of our participants, leading to a significantly higher KE voluntary activation after the WBV training.

Interestingly, we found that WBV training also improved the maximal voluntary isometric knee flexion (+22.7%; pooled values before and after the fatiguing exercise) in comparison to the SHAM group. Although knee flexion strength gains were of a smaller magnitude (i.e., 8.2% to 13.2%), this observation has been previously reported after WBV training programs of similar duration [4,31]. During standing WBV exercises on the platform, the vibration stimulus propagates through the body [32] and the knee flexor muscles stabilize the knee joint during the isometric squat positions. During a single session of WBV, it was evidenced that the knee flexor muscle electromyographic activity significantly increased in comparison with the SHAM conditions [32,33,34]. Therefore, the WBV stimulus delivered here might contribute to the maximal voluntary isometric strength gains observed during knee flexion. Although it was not measured, it can be speculated that the present WBV training program might have increased the voluntary activation of the knee flexor muscles, as was observed for the KE.

Although WBV training tended to increase the KE MVIC (+6.25% between PRE and POST values measured before the fatiguing exercise), no significant modification of the 50% MVIC target was observed, indicating that the absolute target value to reach during the fatiguing exercise was similar between PRE (111.5 N.m) and POST (114.5 N.m) training periods. After the 6-week training period, we observed that the time-to-exhaustion significantly increased (+34%) after the WBV training in comparison to the SHAM training. This result aligns with the 36% greater endurance time reported during a static task of the plantar flexor muscles after four weeks of WBV training [20]. In our study, although MVIC improvements can partially account for the increased time-to-exhaustion after WBV training, other endurance adaptations associated with the WBV stimulus may have also contributed. For example, after a single WBV session performed with synchronous vertical platforms, energy expenditure [35,36], skin blood flow [37], and blood flow velocity [38] were increased in comparison to the same session without WBV. In the same vein, arterial stiffness was also reported to decrease after a session of WBV [39]. Taken together, it could be suggested that these acute WBV observations could explain the longer time-to-exhaustion observed after our repetitive WBV training sessions through a better capacity of the vascular system to carry oxygen to the exercising muscles. Nevertheless, in comparison to the same training without WBV, no vascular adaptations were reported in the healthy participants after a 6-week WBV program performed on a side-alternating platform [40]. In addition, according to an animal study [21], it was observed that the relative number of fast, fatigue-resistant, motor units increased after three months of WBV training. Although speculative, it can be proposed that a similar adaptation could have occurred in the KE of our participants after the 6-week WBV training program. Another possible mechanism mediating the increased time-to-exhaustion could arise from specific hormonal responses to the WBV exercise. For example, it has been previously suggested that testosterone could have a protective effect against fatigue development [41]. This rapid testosterone action during exercise, known as a non-genomic mechanism [42], could counteract fatigue by compensating the muscle contractility impairments occurring during the fatiguing exercises [43]. In this regard, the WBV studies performed with the synchronous vibration platforms evidenced that testosterone concentration was increased immediately in the upper [44] and lower limb muscles [45] in the healthy male participants. However, this immediate elevation of testosterone concentration was not reported after the first and last WBV session of a 9-week training program [3]. Although the heterogeneity of the experimental designs may account for these discrepancies, it can be speculated that the repetitive, non-genomic, hormonal responses occurring after each WBV session may contribute to the lengthier time-to-exhaustion observed after our WBV training. Future WBV studies using synchronous vertical platforms should examine the chronic effects of WBV training on vascular, hormonal, and motor unit adaptations.

This study is the first to report an attenuation of KE neuromuscular fatigue (i.e., MVIC decreases after the fatiguing exercise), induced by a submaximal fatiguing exercise maintained to exhaustion after the WBV training compared to pre-training (i.e., −6.2% vs. −14.8%, respectively). More interestingly, this reduced neuromuscular fatigue was evidenced in conjunction with an increased time-to-exhaustion. To our knowledge, only one animal study reported a similar observation during a fatigue test (i.e., less muscle force reduction) in fast, fatigue-resistant motor units after 3-months of WBV training [21]. Overall (regardless of the group and training), the fatiguing exercise led to PCP impairments, which is an indicator of contractile protein sensitivity to Ca^2+^ [46], suggesting that peripheral factors contributed to task failure [47]. Collectively, along with the above-mentioned non-genomic action of testosterone, particularly occurring in fast-twitch fibers [42], it can be assumed that cross-bridge kinetics were maintained through increased Ca^2+^ levels and mobilization [42,43,44,45]. In addition, the sum of the KE RMS/M_max_ values was also decreased after the fatiguing exercise, but only in the WBV group regardless of the training measurements. This result can be interpreted as a reduced descending central drive from the supraspinal centers to the KE and could contribute to the MVIC loss. While the KE RMS/M_max_ values decreased, no change was observed in the voluntary activation after the fatiguing exercise. Although surprising, voluntary activation is a more global measurement of the KE and may involve the participation of other muscles than those measured in the KE RMS/M_max_ values. In addition, the increased voluntary activation observed after our WBV training could contribute to the overall absence of its impairment after the fatiguing exercise. Consequently, although KE neuromuscular fatigue seemed to involve peripheral and central factors, their exact contribution to the increased time-to-exhaustion and to the reduced MVIC loss, induced by the fatiguing exercise after the WBV training, needs further investigation.

Some limitations of the present investigation should be mentioned. First, the number of participants included in each group in our investigation is relatively small. Although the sample size of the present study is similar to the number of participants included in previous WBV studies [2,3,4,6], our small sample size could partly explain the tendency of the KE MVIC gains observed in our population of physically active males. Second, since only healthy, physically active, males were included in the present investigation, the generalization of the results to healthy, physically active, females or other populations is not possible. Third, although the participants were required to maintain their usual physical activity level during the experimental period, their daily physical activity level was not monitored with accelerometers, for example. Hence, within the limits of the data provided by accelerometers regarding the intensity of the exercise performed, it cannot be ascertained that the participants of the WBV and SHAM groups had a similar total volume of exercise over the 6-week period (except for the resistance training program of the present investigation that was matched between groups). Last, the results obtained here depend on the type of WBV platform used (i.e., synchronous vertical) and the effective acceleration delivered. Considering the variability in an individual’s response to a WBV stimulus [30,45], it is of interest to confirm the concomitant increase in time-to-exhaustion and the attenuated neuromuscular fatigue reported here with a larger sample size.

The WBV literature still needs more studies investigating the underlying mechanisms (e.g., neural, muscular, vascular, hormonal, etc.) contributing to the chronic strength adaptations after the WBV training programs in both male and female participants. These studies should be conducted according to formulated recommendations [26,27,28,29], especially regarding the measure of the effective acceleration delivered by the platform. Nevertheless, the present investigation emphasized relevant outcomes to cope with fatigue in clinical practice. To date, some studies have reported that WBV training could reduce and/or limit fatigue in patients with fibromyalgia [48], multiple sclerosis [49], and more recently, in patients undergoing allogeneic hematopoietic cell transplantation [50]. However, in these studies, fatigue was assessed via questionnaires and/or visual analog scales. Therefore, it is of interest to investigate if WBV training programs could attenuate neuromuscular fatigue (i.e., maximal voluntary contraction decline) in these populations or other health-compromised individuals.

## 5. Conclusions

This 6-week WBV training program was undertaken using a synchronous vertical platform, delivering an effective acceleration of ~3.4 g, which led to significant improvements in the KE voluntary activation and tended to increase the KE maximal voluntary isometric contraction. In addition, we demonstrated that the implemented WBV training program was an effective method to increase the time-to-exhaustion during a submaximal isometric fatiguing exercise performed at 50% of MVIC. For the first time, we demonstrated that WBV training reduced the amount of KE neuromuscular fatigue at exhaustion, though the underlying mechanisms accounting for the longer time-to-exhaustion, as well as the relative contribution of peripheral and central factors of this reduced KE neuromuscular fatigue after WBV training, need further investigation.

## Figures and Tables

**Table 1:** Neuromuscular function of the WBV and SHAM groups measured before (PRE) and after (POST) the training period as well as before and after the fatiguing exercise.

			Before the Fatiguing Exercise				After the Fatiguing Exercise			
			KE MVIC	VA	RMS/M_max_	KF MVIC	KE MVIC	VA	RMS/M_max_	KF MVIC
			N.m	%	a.u.	N.m	N.m	%	a.u.	N.m
WBV	PRE	mean	222.89	90.62	0.26	106.94	189.55	86.70	0.21	110.47
		SD	30.58	8.81	0.10	25.55	33.88	10.41	0.12	24.74
	POST	mean	235.17	95.17	0.25	130.05	222.45	91.89	0.21	130.54
		SD	36.62	3.84	0.08	24.14	51.07	9.27	0.06	27.78
SHAM	PRE	mean	208.35	89.63	0.22	90.42	192.23	89.51	0.24	89.29
		SD	32.09	8.50	0.04	17.01	39.85	11.46	0.04	18.98
	POST	mean	213.56	85.37	0.25	100.00	195.56	87.07	0.27	92.15
		SD	37.16	9.77	0.07	18.19	36.33	10.09	0.08	13.46

KE Knee extensor muscles, MVIC Maximal voluntary isometric contraction, VA Voluntary activation of the knee extensor muscles, RMS/M_max_ Sum of the KE RMS/M_max_ values, KF Knee flexor muscles. a.u. Arbitrary unit.

**Table 2:** Electrically evoked contractions of the knee extensor muscles of the WBV and SHAM groups measured before (PRE) and after (POST) the training period as well as before and after the fatiguing exercise.

			Before the Fatiguing Exercise				After the Fatiguing Exercise			
			Pt_S_	Pt_D_	Pt_DPot_	PCP	Pt_S_	Pt_D_	Pt_DPot_	PCP
			N.m	N.m	N.m	a.u.	N.m	N.m	N.m	a.u.
WBV	PRE	mean	31.39	72.10	99.32	1.32	45.52	89.48	96.68	1.12
		SD	8.99	6.80	9.56	0.13	15.65	8.93	13.84	0.13
	POST	mean	38.52	81.01	98.12	1.26	38.68	82.99	93.91	1.13
		SD	10.94	11.63	20.26	0.09	14.39	19.09	22.07	0.12
SHAM	PRE	mean	35.90	80.18	94.38	1.18	37.33	81.20	87.73	1.09
		SD	17.90	20.85	25.01	0.12	11.77	15.42	14.13	0.09
	POST	mean	46.05	88.66	99.35	1.13	42.53	88.84	97.68	1.11
		SD	15.64	21.10	20.41	0.11	15.49	17.56	14.69	0.08

Pt_S_ Maximal peak twitch at rest (1 Hz), Pt_D_ Maximal peak twitch at rest (100 Hz), Pt_DPot_ Potentiated peak twitch (100 Hz), PCP Post-contraction Potentiation. a.u. Arbitrary unit.

## Data Availability

The datasets generated during the present study are available from the corresponding author upon reasonable request.
